# Integration of multiple types of genetic markers for neuroblastoma may contribute to improved prediction of the overall survival

**DOI:** 10.1186/s13062-018-0222-9

**Published:** 2018-09-20

**Authors:** Aneta Polewko-Klim, Wojciech Lesiński, Krzysztof Mnich, Radosław Piliszek, Witold R. Rudnicki

**Affiliations:** 1 Institute of Informatics, University of Białystok, Konstantego Ciołkowskiego 1M, Białystok, 15-245 Poland; 2 Computational Centre, University of Białystok, Konstantego Ciołkowskiego 1M, Białystok, 15-245 Poland; 3 Interdisciplinary Centre for Mathematical and Computational Modelling, University of Warsaw, Pawlińskiego 5A, Warsaw, 02-106 Poland

**Keywords:** Neuroblastoma, Feature selection, Machine learning, Random forest, Copy number variation, Gene expression, Synergy

## Abstract

**Background:**

Modern experimental techniques deliver data sets containing profiles of tens of thousands of potential molecular and genetic markers that can be used to improve medical diagnostics. Previous studies performed with three different experimental methods for the same set of neuroblastoma patients create opportunity to examine whether augmenting gene expression profiles with information on copy number variation can lead to improved predictions of patients survival. We propose methodology based on comprehensive cross-validation protocol, that includes feature selection within cross-validation loop and classification using machine learning. We also test dependence of results on the feature selection process using four different feature selection methods.

**Results:**

The models utilising features selected based on information entropy are slightly, but significantly, better than those using features obtained with t-test. The synergy between data on genetic variation and gene expression is possible, but not confirmed. A slight, but statistically significant, increase of the predictive power of machine learning models has been observed for models built on combined data sets. It was found while using both out of bag estimate and in cross-validation performed on a single set of variables. However, the improvement was smaller and non-significant when models were built within full cross-validation procedure that included feature selection within cross-validation loop. Good correlation between performance of the models in the internal and external cross-validation was observed, confirming the robustness of the proposed protocol and results.

**Conclusions:**

We have developed a protocol for building predictive machine learning models. The protocol can provide robust estimates of the model performance on unseen data. It is particularly well-suited for small data sets. We have applied this protocol to develop prognostic models for neuroblastoma, using data on copy number variation and gene expression. We have shown that combining these two sources of information may increase the quality of the models. Nevertheless, the increase is small and larger samples are required to reduce noise and bias arising due to overfitting.

**Reviewers:**

This article was reviewed by Lan Hu, Tim Beissbarth and Dimitar Vassilev.

## Background

The current study is the answer to the CAMDA Neuroblastoma Data Integration Challenge (camda.info). The goal of the challenge was the exploration of the opportunities given by the availability of different types of molecular data for improving prediction of patient survival in neuroblastoma.

Neuroblastoma is a cancer manifesting in early childhood. It displays a heterogeneous clinical course and a large fraction of patients with neuroblastoma will eventually enter metastasis and have a poor outcome. Accurate identification of the high-risk group is critical for delivering an appropriate targeted therapy [1]. Currently, the prognosis is based on clinical stage and age of the patient [2]. However, research towards inclusion and integration of genomic data with expression profiles and traditional clinical data is actively pursued in the field [3]. In particular, the effort towards establishing a connection between clinical outcome and gene expression has been recently the subject of a multinational project involving multiple bioinformatical and analytical laboratories [4], where gene expression profiles of 498 patients were examined using both microarrays and RNA sequencing. Within the CAMDA Neuroblastoma Challenge this data has been accompanied with previously generated data relating copy number variation (CNV) for the subset of patients consisting of 145 individuals [2, 5–7]. The clinical data was available for all patients, including survival time, classification to the low- or high-risk subset, as well as sex.

Most of the data in the challenge was already used in the study aiming at comparison of utility of RNA-seq and microarray data sets for prediction of the clinical endpoint for neuroblastoma. What is more, the goal of the CAMDA challenge is a logical extension of goals pursued in that study. Therefore, the current study is based on general methodology proposed by Zhang et al.

However, the detailed analysis of the results obtained in that study shows that significant modifications in the methodology are required. In particular, the design of the Zhang et al. did not allow for the robust and reproducible estimate of predictive power of different models. The study was performed using a single split of data between training set, used to develop models, and validation set, used for assessing the quality of predictions. Six independent groups developed models using data from the training set, the quality of which was then assessed on the validation set. Sixty models using different approaches and different sets of variables were built for each of the six different clinical endpoints. The predictive power of each model was also estimated using cross-validation on the training set. The metric of choice was Matthews Correlation Coefficient (MCC) [8] which is a balanced measure of the predictive power of a binary classifier. In comparison with the simple accuracy measure, it assigns greater weight to prediction of minority class for unbalanced data sets.

Unfortunately, the predictive power of models measured on the training set was not correlated with the predictive power measured on the validation set. Only for models predicting the sex of a patient, correlation between the quality of the model measured on the training set and that measured on the validation set was 0.41, which is statistically significant, if not very high. Nevertheless, this endpoint is not clinically interesting and it was used in the study merely as a reference representing a very easy modelling target.

For all other clinical endpoints correlations between MCC obtained in cross-validation and MCC obtained on validation sets are very small, confined to a small interval between -0.1 and 0.11. What is more, the variance of MCC obtained both on training and validation sets was very high. For example, the following results were obtained for the overall survival: the mean MCC on the training set and validation set for 60 models was 0.48 and 0.46, and 95% confidence interval is (0.46,0.51) for the former and (0.45,0.49) for the latter. The high variance and lack of correlation between predictive power of the models obtained on the training and the validation sets precludes definitive statements about overall superiority of one classifier over another, including comparison of relative merits of different data sets used to build the classifiers.

Since the main goal of the current study is to examine whether integrating multiple lines of experimental evidence can improve the quality of predictive models, high confidence in robustness of results is crucial. For this purpose, we propose a protocol that gives robust results that are well correlated between training and validation sets. The protocol is based on an extensive cross-validation and utilises four methods for selecting informative features used for model building. We apply this protocol to examine the relative utility of different data sets for predicting a single clinical endpoint, namely the overall survival. Finally, we apply the same protocol to examine whether models that utilise informative variables from more than one data set have a higher predictive power in comparison with the models utilising information from a single data set. The protocol includes a feature selection step. Hence, it allows to explore differences and similarities between genes selected as most informative from three independent experimental methods.

## Methods

The single split of data between training set and validation set is not sufficient for robust estimate of performance of the machine learning model on external data. Modelling procedure that includes variable selection and model building is prone to overfitting in both steps. The variable selection finds variables that are informative due to the true relationship with the decision variable, however, the strength of the relationships is modulated by random fluctuations. Hence, variables that appear as most relevant in the training set may be weaker in the validation set. Since the fluctuations in the validation set are independent from the fluctuations in the training set, one can expect that the predictive quality of the model should be weaker on the validation set. The analysis of [4] shows that this decrease is not uniform. On the contrary - the decrease of the predictive power between training and validation set is correlated with the latter. The models that were overfitted the most pay the highest penalty.

The problem is unavoidable when only a single split between the training set and the validation set is used for evaluation of the model performance. The only solution is to switch focus from the individual model to the entire model building pipeline. In particular, this pipeline should encompass the crucial step of selecting variables that will be used by the classification algorithm to build a model. A standardised and reproducible modelling strategy should be used for numerous independent splits of data, and performance of the strategy should be measured as an average over sufficiently large number of tests.

To this end, we propose the following protocol:

1. identification of all informative variables in all data sets generated with different experimental techniques,
2. selection of a limited subset of the variables in each data set,
3. optional merging of data sets from different experiments,
4. building predictive models using machine learning algorithms.

The verification of the predictive power of the protocol is performed with the help of a cross-validation procedure. The model building step is performed using entire available data and the verification of the robustness is performed using two-tiered cross-validation. The first step, namely identification of informative variables, aims at two tasks: one is the removal of variables that are non-informative from consideration, another is producing ranking of relevant variables. All data sets in the study are very high-dimensional. Removal of irrelevant variables transforms the problem to a more tractable one.

In all cases, with the exception of CNV data set, the number of genes that carry information on the decision variable is still much too large for modelling. Therefore, a very simple selection of variables is applied, namely selecting N variables with highest importance score, for model building. This is a naive method, but reasoning is that all non-redundant variables should be included when sufficiently large number of variables is considered. The maximal number of variables considered was set at 100 due to our previous experience with gene expression data and preliminary experiments with the current data sets. Both suggest that performance of the predictive models either stabilises or even starts to decrease when number of variables included in the model is larger than that.

### Data

The data sets used in the current study were obtained from the CAMDA 2017 Neuroblastoma Data Integration Challenge (http://camda.info). Genetic information was collected using three different experimental techniques, namely profiling of gene expression (GE) by means of microarray, RNA sequencing, as well as analysis of copy number variation profiles using array comparative genomic hybridization. The data collection procedures and design of experiments were described in the original studies [2, 4–7]. The data is alternatively accessible in Gene Expression Omnibus (https://www.ncbi.nlm.nih.gov/geo/) with accession number GSE49711 (gene expression) and GSE25771, GSE35951, GSE45480, and GSE56109 (copy number variation). The following data sets are available:

1. 39 115 array comparative genomic hybridization (aCGH) copy number variation profiles, denoted as *CNV*,
2. 43 349 GE profiles analysed with Agilent 44K microarrays, denoted as *MA*,
3. 60 778 RNA-seq GE profiles at gene level, denoted as *G*,
4. 263 544 RNA-seq GE profiles at transcript level, denoted as *T*,
5. 340 414 RNA-seq GE profiles at exon-junction level, denoted as *J*.

Data for 498 patients is available in the *MA, G, T* and *J* data sets, whereas the *CNV* data set is limited to 145 patients. Therefore, a full analysis is performed for 145 patients and a separate analysis is performed for 498 patients using four data sets. The data sets are further referred to as *X-number*, where *X* corresponds to data set, and *number* is either 498 or 145. For example, MA-145 denotes MA data set limited to a cohort of 145 patients. Both cohorts are unbalanced. There are 393 survivors versus 105 non-survivors (21% of non-survivors, 79% survivors) in the larger cohort. The smaller cohort is slightly less unbalanced with 107 survivors versus 38 non-survivors (26% of non-survivors, and 74% survivors).

#### Statistical properties of gene expression and CNV data

Data sets used in the current study correspond to two different biological phenomena, measured using 5 different experimental techniques resulting in different statistical properties of their distribution. Nevertheless, they can be analysed using the same general protocol. In all cases we look for the difference between samples taken from two populations. In the case of gene expression we look for the differentially expressed genes, whereas in the case of CNV data sets we look for genes that have different number of copies in two populations.

Gene expression was measured by RNA-seq as well by microarray hybridisation, whereas CNV variation was measured by two-channel microarrays. Despite different biological phenomena under scrutiny, signal from both microarray experiments has similar properties. In both cases the signal is transformed to logarithmic scale. In this scale the signal distribution is approximately normal in most cases. The normality was tested using two tests, Kolmogorov-Smirnov (KS) [9] and Shapiro-Wilk (SW) [10], implemented in R. Both tests were performed separately for each decision class (survivors/non-survivors). For the MA-145 data set, the less strict KS test accepted hypothesis of normality in 88% of cases, while the more strict SW test confirmed normality in 51% of cases (both numbers are given for the more numerous class, slightly higher values were obtained for the less numerous one). In the case of CNV data set, the corresponding numbers are 96% for KS test and 48% for SW test.

The signal from gene expression measurement obtained by means of RNA-seq has markedly different statistical properties than one obtained from the microarray measurements. In the case of microarrays, the physical signal is an intensity of fluorescence from probes hybridised to gene-specific sondes. In the case of RNA-seq, the raw signal is a number of reads that map to a gene. It is then preprocessed in a RNA-seq specific pipeline and normalised. The RNA-seq data available for CAMDA challenge was preprocessed by the Magic-AceView pipeline (MAV), based on the Magic analysis tool [11] (https://bit.ly/2K0jkwi), see Zhang et al. for details [4]. The final expression signal is a logarithm of the signal normalised to FPKM units. The gene expression signal measured by RNA-seq is not close to normal distribution for most genes. Only 9% of variables are normally distributed according to the SW test and 38% pass the KS test.

#### Data preprocessing

All datasets were preprocessed before they were used in analysis. In the first step the data sets were carefully inspected manually. It turned out that CNV data in particular required manual curation. The CNV measurements were performed in 7 laboratories, with two different Affymetrix platforms. Each laboratory has used slightly different file formats, with varying numbers of rows and columns. In some cases the reference and test samples were marked with different fluorescent markers. The manual curation involved selection of a common set of probes and mapping results to the single signal direction. After initial manual inspection and curation, the variables with more than 10% of missing values were removed from the data sets. Then for each variable that still contained missing values, they were replaced by the median value. Finally, the effects of confounding values were examined and removed with the help of SVA package [12] from Bioconductor [13] (https://bit.ly/2yod7FC). The MA-498, and RNA-seq data sets have been preprocessed earlier in the original study, hence there was no need for the additional preprocessing. In particular no batch effects were discovered with SVA package. The scripts for data preprocessing are available upon request.

### Identification of informative variables

In the first step of the procedure, we aim to identify all relevant variables [14, 15] with the help of three methods: t-test, simple univariate information gain, and two-dimensional conditional information gain.

**T-test** In the first approach we perform a standard test of difference of means for two populations corresponding to distinct clinical endpoints, namely overall survival and death. Let $\bar {x}_{s}$ be the average value of variable *x* for those subjects who survived and $\bar {x}_{d}$, for those who did not. The tested null hypothesis is equality of two means, $\bar {x}_{s}=\bar {x}_{d}$, and the test statistic is obtained as:

$t = \frac {\bar {x}_{d} - \bar {x}_{s}}{\sqrt {{\frac {V_{d}}{n_{d}} + \frac {V_{s}}{n_{s}}} }},$

with analogous subscript annotations for variance *V* and population size *n*. Since multiple tests are performed, the Hochberg correction [16] is applied to *p*-value required to reject the null hypothesis.

**Information gain** We have recently developed a methodology for testing relevance of variables using information theory [15, 17]. To identify variables *x*∈*X* which exhibit statistically significant influence on a response variable *Y* we use the conditional mutual information between *Y* and *x* given the subset S: *S*⊂*X*:

*IG*(*Y*;*x*|*S*)=*H*(*x*,*S*)−*H*(*Y*,*x*,*S*)−[*H*(*S*)−*H*(*Y*,*S*)]

where H(x)denotes the information entropy of the variable *x*.

*IG*(*Y*;*x*|*S*) can be interpreted directly as the amount of information about the response variable *Y*, that is contributed by the variable *X* to the subset *S*. It is always non-negative and becomes zero when the variable contributes no information to the subset.

It is worth noting that in the univariate case, i.e. if the subset *S* is empty, *IG*(*Y*;*X*|*S*) reduces to the mutual information of *Y* and *X*, commonly used to test the statistical association between the variables.

*IG*(*Y*;*X*|*∅*)=*IG*(*Y*;*X*)

The conditional mutual information has been already used in the context of minimal-optimal feature selection, see for example [18–21]. However, it has not been used for identification of the synergistic relevant variables. For non-empty subset *S* the exhaustive search over all possible tuples of variables $x_{i_{1}},\ldots,x_{i_{k}}$ is performed. The maximal information gain

*IG*_*max*_(*x*)= max*S*⊂*X*(*IG*(*Y*;*x*|*S*))

is a measure of relevance of variable *x*. Statistical significance of *IG*_*max*_(*x*) can be assessed using extreme value distribution of *IG*_*max*_ computed for all variables in the exhaustive search.

The dimensionality of the exhaustive search is limited both by the need for adequate sampling of data and by computational resources. Two cases are explored in the current study, namely *S*=*∅* and |*S*|=1. In the first case, labeled as IG-1D, a simple univariate search for relevant variables is performed, whereas in the second one, labeled as IG-2D, for each tested variable *x*_*i*_∈*X* all pairs with *x*_*j*_∈*X* are examined.

### Selection of the feature subset

In most cases relevant variables identified by the filters mentioned in the previous section are too numerous to be useful for further analysis. Therefore, a procedure for selecting a subset of variables is necessary. To this end, we sort variables according to the *p*-value of the relevance score and select top *N* variables, *N*∈{10,20,50,100}. In the case of t-test one more set of relevant variables is obtained by building the lasso regression [22] model for the response variable and selecting variables present in *N*-dimensional models, with *N*∈{10,20,50,100}.

No additional selection was performed for the subset of top *N* features, in particular no removal of redundant or correlated variables. The initial tests have shown that removal of correlated variables has generally no effect on the quality of final models. In some cases, the quality was slightly improved, but for some others it decreased with no measurable net effect overall.

### Predictive models

Predictive models were built using selected informative variables with the help of Random Forest classification algorithm (RF) [23] implemented in the *randomForest* library [24] in R [25]. Random Forest is a general purpose machine learning algorithm for classification and non-parametric regression that is widely used across multiple disciplines. It is an ensemble of decision trees. Each tree is built using a different sample of data, and each split of a tree is built on a variable selected from a subset of all variables. The randomness injected in the process of tree construction has two effects. On one hand, it significantly decreases classification of the individual tree. On the other, it decorrelates individual classifiers and helps to decrease overfitting. What is more, for each tree there is a subset of objects, that were not used for construction of this tree, so called out of bag (OOB) objects. This allows for an unbiased estimate of the classification error and variable importance. For each object there are several trees that did not use it for model building, hence it is an OOB object for these trees. To estimate the classification error all trees predict the class for their OOB objects. The predictions are then pooled together and the class for each object is assigned by vote of all OOB trees. This prediction is then compared with the true class of each object to estimate quality of the model. Quality estimates based on this procedure are called OOB estimates.

Random forest has many applications in bioinformatics, for example in gene expression studies [26, 27], in discovering protein-protein interactions [28, 29], or in genetic association studies [30–32]. In a recent comparison of 179 classifiers from 17 families, performed on 121 data sets, classifiers from the RF family have shown the best and the most robust performance [33]. In particular, the performance of RF classifiers was usually very close to the best achieved for a particular problem. Only in a handful of cases was it significantly worse than the best one.

The alternative algorithm that is frequently used for analysis of gene expression data is Support Vector Machine (SVM) [34], which usually gives very good classification results for this type of data. The comparisons between the two methods have first shown a slight advantage of Random Forest for analysis of gene expression [26]. These findings were not confirmed in another study [35], which has shown a slight advantage of SVM. Nevertheless, both algorithms are still used for building predictive models for gene expression, and some new reports show a relative advantage of Random Forest over SVM on various sets of problems [36, 37].

Two properties of Random Forest classification algorithm make it particularly suitable for the current study. The first one is a natural propensity of Random Forest for discovering complex nonlinear and non-continuous relations in data. This property is ideally suited for the goal of the study, namely a search for possible non-linear synergies between variables describing different biological phenomena. Indeed, our own experience with Random Forest classifier shows that in the presence of highly linear interactions between variables it has significantly better accuracy than SVM [38]. Another advantage of RF for the current study is the low sensitivity of results to the selection of parameters. Random Forest has few tunable parameters, and the results are usually only slightly dependent on them. In particular, the two most important parameters are the number of trees in the forest and the number of variables tested when a split is generated. In comparison, the performance of SVM is critically dependent on the selection of the kernel function suitable for the particular dataset. What is more, tuning of the parameters of the kernel function is usually required, which is often a computationally intensive task. In our approach all tuning of parameters would be performed within a cross-validation loop. The application of RF with default parameters allows to avoid this computational burden.

### Comparisons between models

The predictive power of each model is estimated using Matthews correlation coefficient (MCC) [8], following the approach proposed by Zhang et al. [4]. MCC is a measure proposed for estimation of classification performance for imbalanced data sets. It is a measure of the predictive power of models, obtained as a geometric mean of informedness and markedness of a model computed from the confusion matrix, see [39] for a thorough explanation. It is an unbiased measure that treats both classes with equal weight and is generally recommended for measuring quality of machine learning models [40].

Models are compared using three approaches that differ in the level of independence between training and test set. In the original setup of Zhang et al. the full data set was split randomly in two parts - the training set used for model building and test set used for evaluation of predictive power. Additionally, the predictive power of the models was evaluated in 10 repeats of cross-validation performed on the training set. Unfortunately, this setup has several significant drawbacks. Firstly, the models are built using only half of the available data. While this may not be a problem for large data sets, the smaller data set in the current study contains only 38 subjects in the minority class. This is a small sample, that may significantly limit the quality of the model. What is more, the performance on the test set depends strongly on the single split of data between training and test set. The more or less fortuitous fit of the model to the particular split is a single most significant factor influencing the results in such a design, and therefore it is useless for comparison of different modelling strategies.

Instead, we propose a three-stage setup for comparison of modelling strategies. In each stage a different balance between bias and error is obtained by using a different split between training and test sets for different steps of model building.

**Minimum error – maximum bias:** In the first stage all available data is used for the entire modelling process - both for feature selection and for model building. This stage gives the most optimistic estimate of the quality of the models. Due to the construction of the Random Forest model, a nearly independent estimate of the model quality is still possible even at this stage by means of the the out of bag (OOB) error estimate.

**Intermediate bias and error:** In the second stage the feature selection step is performed once, using all available data. Then, modelling is performed using *k*-fold cross-validation. Multiple repeats of cross-validation procedure are performed to alleviate the dependence of results on a single split of data. In each repeat the data set is independently split into *k* parts. To preserve the proportion of minority and majority class in each part, both classes are split separately and then merged. Then the following procedure is applied:

1. build a training set using *k*−1 parts, assign the remaining part as a test set,
2. build a model on the training set,
3. evaluate model performance on the training set,
4. evaluate model performance on the test set.

The performance estimate is obtained as an average over all independent models.

The second stage allows to estimate the size of two possible effects. The first one is a possible difference of predictive power between OOB and cross-validated estimate. The second one is a possible decrease of predictive power due to decreased size of the training set in comparison with the entire sample. It can be observed as decreased OOB estimate of MCC in the second stage in comparison with the first stage.

**Minimum bias – maximum error:** In the third stage the entire modelling procedure, including the feature selection step, is performed multiple times within *k*-fold cross-validation scheme. Within each repeat the training and test data sets are obtained identically to the previous stage. Then, the following procedure is applied in each iteration of the cross-validation loop:

1. build a training set using *k*−1 parts, assign the remaining part as a test set,
2. perform feature selection procedure using data from training set,
3. build a model on the training set,
4. evaluate model performance on the training set,
5. evaluate model performance on the test set.

This stage allows to estimate the influence of overfitting due to feature selection process. The possible difference between OOB and cross-validated estimate of MCC of models may arise due to the combination of three effects

- overfitting due to feature selection,
- overfitting in the OOB estimate of error,
- decrease of predictive power due to smaller sample size.

The two latter effects can be accounted for by using estimates from stage two, hence, any additional effect will be due to feature selection. What is more, the average predictive power obtained by this full cross-validation is our best conservative estimate for the predictive power on new subjects.

### Aggregation of data sets

One of the goals of the current study is to examine whether merging information from different technologies (microarray and RNA-seq for gene expression) or pertaining to different biological phenomena (copy number variation and gene expression) can improve our predictions of clinical endpoints for neuroblastoma. To this end, we first identified informative features in all experiments and then created data sets that include relevant features from all pairs of experiments. Then Random Forest models were built on these data sets. Results and predictive power of models built on different features was compared.

We have performed preliminary tests of an alternative procedure where pairs of data sets were merged into a single data set and then feature selection algorithms were applied on a joint data set. It is worth noting that such a procedure has lower sensitivity for univariate methods, due to larger number of variables used in Bonferroni correction, and it cannot change ranking of variables from the same data set. On the other hand, synergies between data sets should be discovered by IG-2D method. Unfortunately, no significant synergies were found when analysis was performed in this way neither between data sets representing different experimental techniques for measuring gene expression nor between gene expression and CNV data sets. Therefore, this alternative procedure was not pursued further.

## Results

### Informative variables

Informative variables were identified for each data set separately. All three filtering methods discovered numerous informative variables in gene expression data analysed with microarrays and various RNA-seq protocols. The summary of the findings is presented in the Table 1. The number of informative variables in these data sets varies between eight hundred identified by IG-2D filter for microarray data in small cohort, to nearly fifty five thousand identified also by IG-2D filter for transcript data in the larger cohort. Two clear trends can be observed in the data. Firstly, there is a dramatic gap in sensitivity of filters between the two data sets, in particular for both filters based on information theory. In the case of t-test increase of number of informative variables increases 5- to 10-fold between smaller and larger cohort, whereas for IG-2D filter the increase is 7- to 22-fold. Secondly, the sensitivity of t-test is the highest for all gene expression data sets in small cohort, but is the lowest for larger cohort. This is a mirror image of the IG-2D filter that is the least sensitive for smaller cohort and the most sensitive for larger cohort.

**Table 1:** Informative variables discovered by three filtering methods in all data sets

	Data set								
	CNV	MA	G	J	T	MA	G	J	T
Variables	145 subjects				498 subjects				
All	39115	43349	60778	340414	263538	43291	60778	340414	263538
Used ^∗^	39114	43349	40660	340414	208856	43291	41104	340414	208058
T-test	5	1152	1096	2738	3726	6420	8180	37011	38324
IG-1D	25	900	1008	1825	2844	6364	9690	36915	46169
IG-2D	37	807	878	1457	2445	11307	11243	44927	54987

^*^Multiple markers in genes and transcript series of RNA-seq data are incomplete, with data missing for most patients. Only markers for which at least 50% of records is non-zero for both decision classes were included in the study

The only exception is the copy number variation data, where the number of informative variables varies between 5 for a t-test and 37 when filter based on pairwise interactions information is used. What is more, the three methods identify rather similar sets of variables for microarray data, whereas divergent sets of variables are obtained for CNV data, see Fig. 2.

This number of informative variables in gene expression data is certainly too large to be useful and a procedure for selecting variables for building predictive models is required.

#### Informative variables for 145 subjects

The main focus of the CAMDA experiment is on the integration between data obtained with the help of different technologies, such as measuring gene expression using microarrays and RNA-seq, or relating to different biological phenomena, such as studying copy gene expression and genetic variation. This analysis can be performed only on the smaller cohort, hence, the more detailed analysis was focused on this subset of data. The number of variables deemed relevant by all filtering methods is much too large for detailed analysis and for model building, hence, we limited the analysis to fifty most important genes identified in MA-145, G-145 and CNV data sets. Two gene expression data sets were selected for the analysis due to better performance of predictive models built on these data sets in comparison with those built on J-145 and T-145. The examination of modelling results reveals that models utilising 50 variables usually give predictions as good, or nearly as good as those built using 100 variables, and significantly better than those built using 20 variables, hence, this number was selected for analysis. Since the number of relevant genes is smaller then that number for CNV data set, all genes were examined for this data set.

In particular, we examined the following questions:

1. what genes are identified as most relevant?
2. to what extent sets of most informative genes in gene expression data are similar across technologies and across filtering methods?
3. which genes are consistently shown as most relevant for each technology?
4. are the genes indicated as most relevant in CNV data set also relevant in gene expression data?

A clear and simple answer may be given to the last question. None of the genes identified as relevant in CNV data set, were identified as relevant in MA-145 or G-145 data set, hence the copy number variance is not reflected in the most important gene expression levels.

##### Gene expression

Microarrays and RNA-seq don’t agree very well on which genes are most informative for the overall survival, see Table 2. The number of genes identified by both technologies within top 50 genes with the help of at least single filter is 16, out of 88 and 100 genes selected to top 50 by at least one filter from MA-145 and G-145 data sets, respectively. Only three genes, namely *PGM2L1*, *SLC22A4* and *PRKACB* were included among highest ranked by all filters in both MA-145 and G-145 data sets. All these genes have been previously identified as important neuroblastoma markers [41–43].

**Table 2:** Informative genes that were identified as most relevant in MA-145 and G-145 data sets

	MA-145			G-145		
	Ranking by			Ranking by		
Gene	T-test	IG-1D	IG-2D	T-test	IG-1D	IG-2D
PGM2L1	1	4	1	1	3	1
SLC22A4	2	20	23	2	13	12
PRKACB	29	1	19	34	4	2
DOC2B	28	7	13	-	37	20
PIK3R1	-	21	9	-	10	6
NTRK1	-	17	8	-	32	32
NRCAM	4	12	-	-	19	-
ALDH3A2	6	-	47	5	-	-
DST	-	-	3	-	43	-
A_32_P30874	32	2	2	-	-	-
Hs23691.1	11	29	4	-	-	-
PLXNA4A	45	3	15	-	-	-
HSD17B3	3	34	-	-	-	-
ACN9	-	-	-	7	5	4
Slartoybo	-	-	-	19	1	5
LOC100289222	-	-	-	18	20	3
Sneyga	-	-	-	-	2	10
SPRED3	-	-	-	3	42	-
Jardarby	-	-	-	4	-	-

All genes that were ranked in top 10 most relevant by any filtering method in either data set are shown. The numbers in each column correspond to ranks achieved by genes in a data set, processed by one of three filtering methods. Genes present in top 50 variables in both data sets are shown first, followed by those present in top 50 only in MA-145 data set, and then by those exclusive in top 50 in G-145 data set

**Fig. 1:**
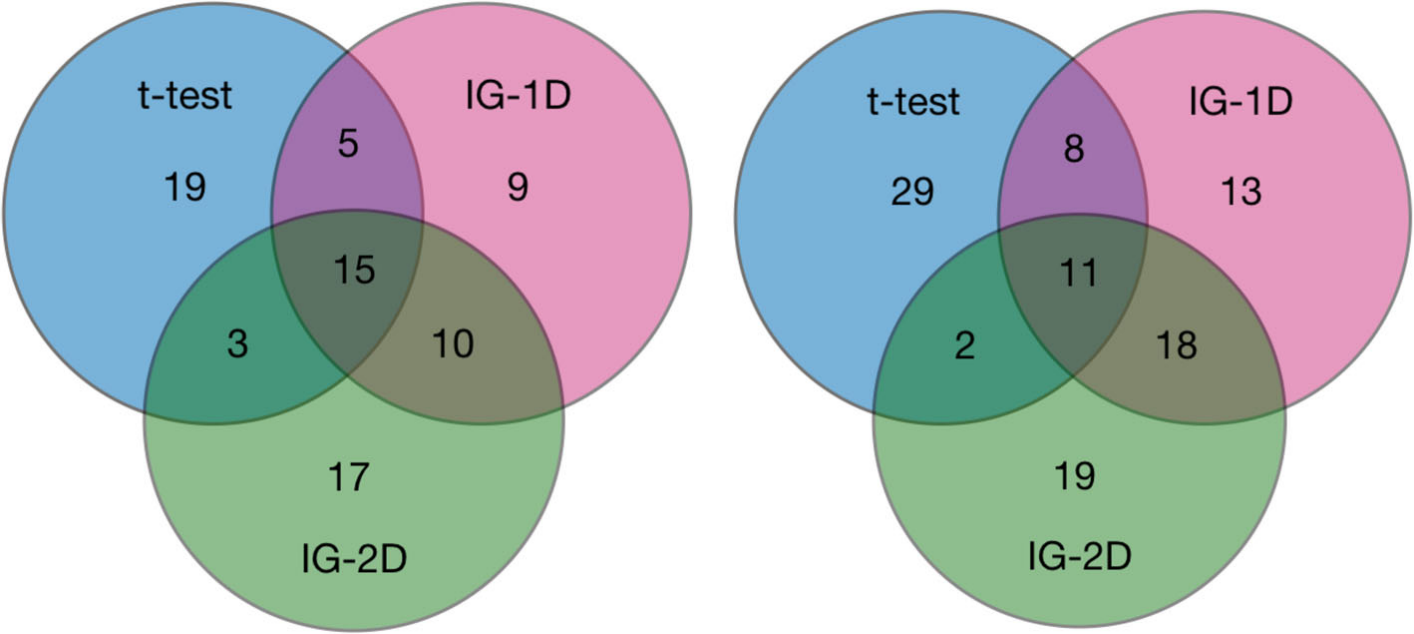
Venn plot for top 50 informative features identified in MA-145 (left panel) and G-145 (right panel) data sets

When single filters are considered separately the t-test and IG-2D each find only 7 genes that are in top 50 most relevant in both technologies. In comparison, IG-1D filter is more consistent since it finds 10 genes that are most important both in MA and RNA-seq data. The agreement between different filters is much higher when measured on the same data set, see Fig. 1.

**Fig. 2:**
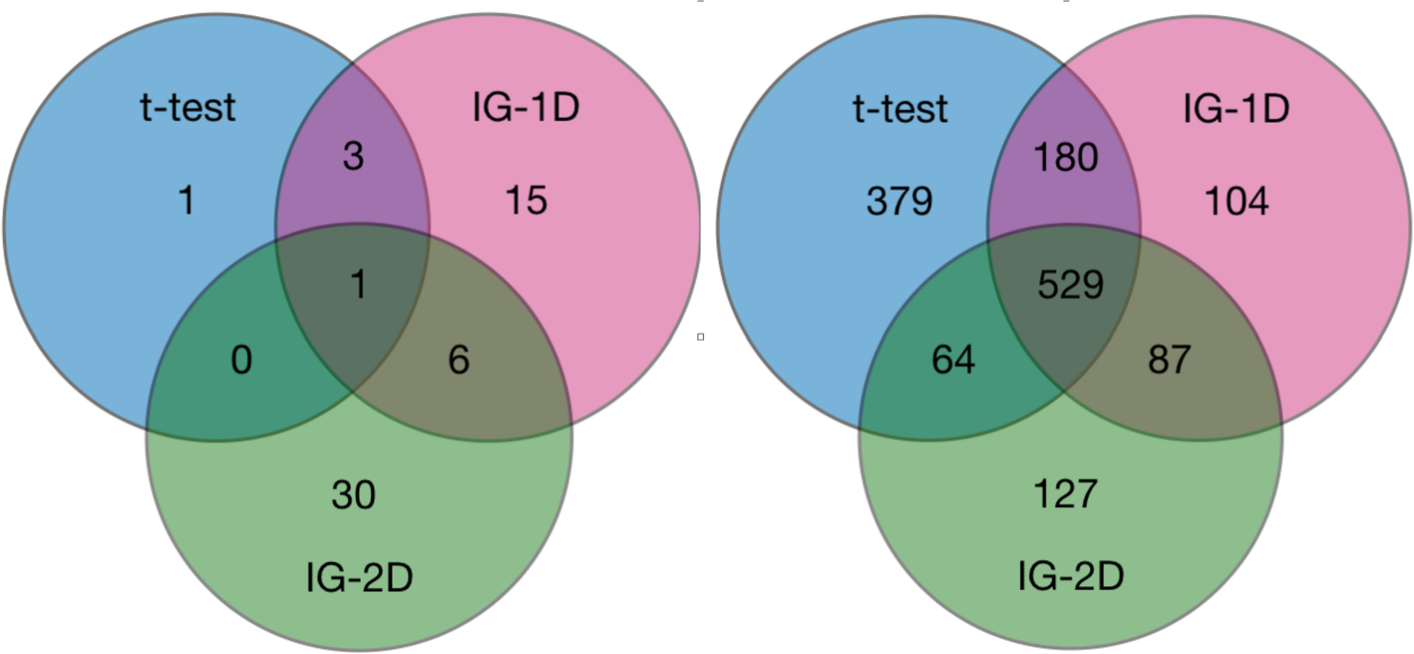
Venn plot for sets of informative features identified in CNV-145 (left panel) and MA-145 (right panel) data sets. There is little overlap between informative features identified by three methods for CNV data. In particular, there is only one variable recognised as relevant by all three filtering methods. The agreement for the gene expression is much higher - for each method the number of variables that is common with at least one other method is larger than 68% of all variables identified as relevant by this method

The two experimental techniques under scrutiny both report the gene expression level, nevertheless the values reported for the same gene by both technologies are different, as discussed earlier. Therefore, direct comparison of the gene expression levels measured by two techniques is not feasible. However, an interesting analysis can be performed by comparing expression level of two groups of genes within each technology separately. To stress that we don’t compare expression levels directly, we use the notion of *signal strength* for this comparison. Interestingly, the average signal strength for genes identified as most relevant for MA-145 and G-145 data sets was identical to the average signal strength for genes identified as most relevant only in MA-145 data set. The signal strength obtained with the microarrays is 12±3 and 11.2±0.6, for the common set and for the set unique to MA-145, respectively. On the other hand, the signal strength measured with RNA-seq for genes identified as relevant only in G-145 data is 12.5±0.7 which is significantly lower than 15±2, that is a signal strength measured by RNA-seq for the common set. This suggests that RNA-seq experiments can reveal strong biological signal in weakly expressed genes better than microarray experiments.

##### Copy number variation

The number of variables identified as relevant in the CNV data set is small in comparison with gene expression data, which can be expected on biological ground. The three filtering methods give widely divergent results, with only one gene identified as relevant by all three methods, see Fig. 2. Five additional genes were identified as relevant by two methods, see Table 3. Interestingly, two highest ranking genes, *ZNF644* and *ZZZ3* code zinc finger proteins. Both genes are involved in regulation of chromatine activity via histone modifications [44, 45]. *TMED5* is involved in vesicular protein trafficking [46], *QKI* is involved in in mRNA regulation [47], and *PLEK2* regulates actin organization and cell spreading [48]. All these biological roles are very plausible for their influence on the progress of neuroblastoma.

**Table 3:** Informative genes that were identified as most relevant in the CNV data set

	Ranking by		
Gene	T-test	IG-1D	IG-2D
ZNF644	2	4	19
ZZZ3	-	1	2
TMED5	-	10	1
PLEK2	1	15	-
QKI	4	16	-
A_14_P117576	5	21	-
KIAA0090	-	-	3
ANKRD13C	-	3	-
FNDC1	3	-	-
GUCA2B	-	-	4
C1orf160	-	-	5
LPHN2	-	5	-

The numbers in each column correspond to ranks achieved by genes processed by one of three filtering methods – t-test, IG-1D or IG2D. All genes that were ranked in top 5 most relevant by either method are displayed

### Predictive models - overview

The predictive models have been built using the three stage approach described earlier. For all data sets a similar pattern of MCC behaviour is observed. The MCC values obtained for all cases where a model is tested using the data set used for feature selection are close to each other. This includes all OOB estimates for stages one, two and three, as well as cross-validated estimate of stage two. On the other hand, significant drop of predictive power is observed in the cross-validated estimate in stage three.

The bias due to feature selection procedure is much higher for data sets describing the smaller cohort. MCC is inflated by 0.10 - 0.13 in this case, compared with the bias of 0.02 for data sets describing larger cohort.

However, the overall results are better for the smaller cohort. The average cross-validated MCC obtained for all models and all data sets is 0.597 and 0.530, for the smaller and larger cohort, respectively, see Table 4.

**Table 4:** Aggregate results for all models based on gene expression

	Data series			
Cohort size	MA	G	J	T
	Max			
145	0.674	0.672	0.606	0.625
498	0.545	0.556	0.543	0.543
	Average			
145	0.634	0.629	0.556	0.569
498	0.535	0.538	0.525	0.524

Maximum and average MCC obtained for all fully cross-validated models built for each data series are displayed for both cohort sizes

The results obtained for RNA-seq and microarrays were very similar for the larger cohort, with slightly lower quality models obtained on J-498 and T-498. On the other hand, for smaller cohort the difference obtained for J-145 and T-145 data sets were significantly worse than those obtained for MA-145 and G-145 data sets. Taking into account that impact of genetic variation is estimated only for the smaller cohort, and that the aim of the current study is exploring integration of various data sets, further analysis of gene expression is limited to MA-145 and G-145 data sets.

It is worth noting that lower quality of predictive models for larger sample is unusual – improved sampling normally leads to better models. Apparently, recruitment of patients to the smaller sample was non-random and included patients for whom predictions were easier. Another interesting effect related to the sample size is the relative quality of models built using MA and G data sets in comparison with those built using J and T data sets. The MCC for models based on J-498 and T-498 data sets is lower by roughly 0.01 than MCC achieved by models built using on MA-498 and G-498. On the other hand, analogous difference for smaller cohort is roughly 0.06. This is probably due to higher noise in junction and transcript data in comparison with direct gene measurements that has dramatic effect on reliability for smaller sample size.

### Results for the smaller cohort

The three-stage setup allows for a precise estimate of the influence of different factors on the quality of predictive models in the cross-validation loop. These effects can be observed by closer examination of results presented in Table 5 and Table 6, where results obtained for MA-145 and G-145 respectively, are presented.

**Table 5:** Model quality measured with MCC coefficient for the MA-145 data set

	OOB				Cross-validation			
FS metod	Top 10	Top 20	Top 50	Top 100	Top 10	Top 20	Top 50	Top 100
Stage 1								
T-test	0.642	0.707	0.700	0.720				
IG-1D	0.753	0.728	0.738	0.736				
IG-2D	0.713	0.747	0.730	0.726				
T-test + lasso	0.744	0.869	0.820	0.822				
Stage 2								
T-test	0.622	0.683	0.693	0.713	0.636	0.698	0.709	0.730
IG-1D	0.732	0.721	0.729	0.730	0.741	0.727	0.737	0.737
IG-2D	0.695	0.734	0.721	0.721	0.699	0.743	0.731	0.729
T-test + lasso	0.732	0.854	0.808	0.809	0.750	0.868	0.831	0.832
Stage 3								
T-test	0.655	0.691	0.714	0.724	0.576	0.606	0.647	0.665
IG-1D	0.735	0.742	0.747	0.748	0.605	0.638	0.661	0.674
IG-2D	0.705	0.721	0.730	0.734	0.609	0.636	0.655	0.670
T-test + lasso	0.780	0.831	0.808	0.820	0.651	0.663	0.648	0.643

**Table 6:** Model quality measured with MCC coefficient for the G-145 data set

	OOB				Cross-validation			
FS metod	Top 10	Top 20	Top 50	Top 100	Top 10	Top 20	Top 50	Top 100
Stage 1								
T-test	0.703	0.720	0.719	0.713				
IG-1D	0.788	0.784	0.801	0.796				
IG-2D	0.793	0.768	0.738	0.763				
T-test + lasso	0.790	0.834	0.862	0.861				
Stage 2								
T-test	0.683	0.709	0.714	0.704	0.692	0.719	0.716	0.712
IG-1D	0.732	0.767	0.766	0.762	0.741	0.782	0.778	0.774
IG-2D	0.729	0.733	0.743	0.759	0.743	0.753	0.756	0.771
T-test + lasso	0.792	0.827	0.848	0.848	0.802	0.840	0.865	0.867
Stage 3								
T-test	0.689	0.713	0.723	0.724	0.590	0.621	0.650	0.653
IG-1D	0.750	0.771	0.774	0.770	0.589	0.626	0.661	0.672
IG-2D	0.738	0.755	0.760	0.755	0.585	0.621	0.650	0.661
T-test + lasso	0.829	0.832	0.853	0.854	0.599	0.661	0.655	0.638

**Fig. 3:**
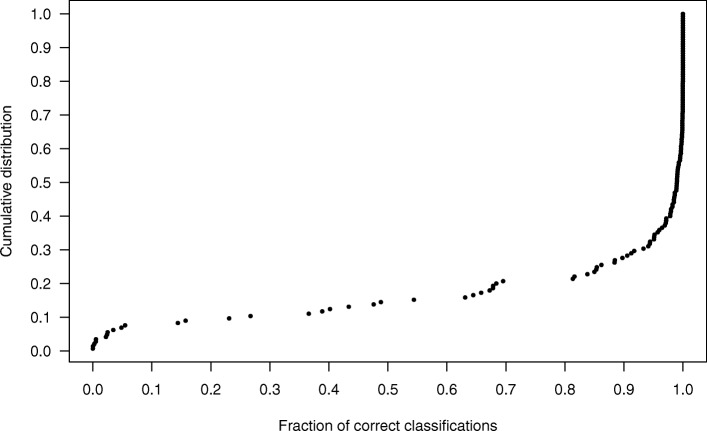
Distribution of fraction of correctly classified objects. For each object the position in y axis corresponds to the fraction of times this object was correctly predicted in cross-validation

**Fig. 4:**
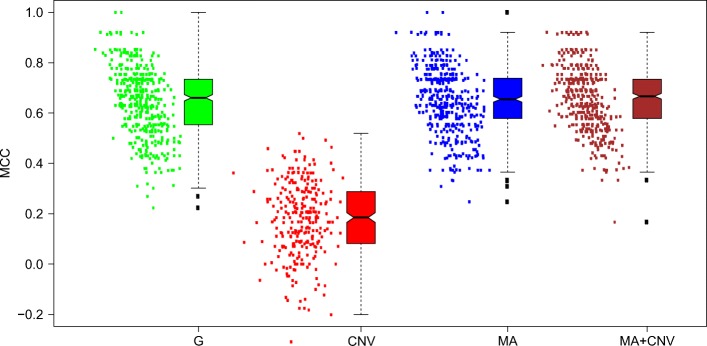
Distribution of MCC obtained in 400 cross-validation runs at the Stage 3 of the modelling pipeline. Each point, representing MCC value obtained for a RF classifier prediction for the validation set in the cross validation loop. Each RF classifier was built on the different training set constructed in the cross-validation loop, using the variables selected as most relevant for a given training set. Values for G-145, CNV, MA-145, and MA+CNV data sets are presented from left to right. Each box-plot represents distribution of points to its left

The first effect that may influence the result is due to the decrease of the training set size in cross-validation. In five-fold cross-validation the training set is 80% of the total. The influence of this effect, is estimated as the difference of MCC measured using OOB estimate in the first and second stage. The decrease of MCC is 0.012 and 0.020 for MA-145 and G-145, respectively. The second effect, often observed for Random Forest classifier, is a slight increase of the predictive power in external cross-validation in comparison with the OOB estimate. This effect may arise since fewer trees (roughly one third) participate in OOB classification of each object in comparison with classification of external validation set. Within the current scheme it can be estimated by taking the difference between MCC obtained in cross-validation and OOB in the second stage. The difference is 0.012 both for MA-145 and G-145 data sets. The third possible effect is overfitting of the classifier due to feature selection. There are two manifestations of this effect. Firstly, the OOB estimate obtained in cross-validation is artificially inflated. This happens because fortuitous selection of objects to the training set may artificially inflate the importance of some variables in it in comparison with the entire sample and allow to build an overfitted model. This effect can be measured as the difference of the OOB estimate of MCC between third and second stage. This difference is 0.012 for the MA-145 data set and 0.011 for the G-145 data set. One should note that since importance of some variables is artificially inflated for the training set, it will necessarily be decreased for the validation set. Hence, the classifiers using this variable will be worse on validation set than on general population. What follows, this effect may artificially bias the estimate of performance downwards. Finally, the sample contains a certain pool of objects that are misclassified with probability higher than 90%, see Fig. 3. The split of these objects between training and validation set has a significant role for OOB and validation set estimate of MCC. In particular, MCC can be very high when none of these objects is in the validation set, and it can be very low, when they are plenty. The excessive estimate of overfitting on validation set is demonstrated by a negative correlation (average correlation coefficient *r*=−0.42) between OOB and cross-validated estimates of MCC, see Fig. 4 (the MCC for this Figure were computed for 500 training- and validation- set pairs).

For each data series the three methods based on selection of *N* variables with highest *p*-value have very similar behaviour. The quality of the model measured using OOB is very similar for all three stages, and similar to the cross-validated measure obtained using single ranking of variables obtained using all available data. However, the predictive power of models developed using fully cross-validated approach is strongly diminished. On the other hand, the models that used variables selected by applying lasso to the feature set identified by t-test are different. For these models a drop of cross-validated measure of MCC is similar for second and third stage. This result shows the extent of quality decrease due to the ranking of variables and the selection of the set. All variables that entered the lasso procedure in the second stage were identical for all 500 individual models. Nevertheless, the selection of variables that produced the best possible model for the training set introduces bias. The strength of this bias is mostly due to the feature selection process itself, not due to the composition of the original set of variables. This is particularly clear for the MA-145 data series.

#### Influence of feature selection methods.

Feature selection has limited influence on the quality of models for MA-145 and G-145 data sets. The overall best result, MCC = 0.674, was obtained using 100 variables selected by IG-1D from the MA-145 data set, however, results obtained with 100 variables selected by IG-2D were within the error margin. The best result obtained for G-145 data set, MCC=0.672, was slightly lower, however still within the estimated error range. It was also obtained using 100 variables selected by IG-1D filter. The models built using variables selected with simple t-test are generally worse than those obtained using either IG-1D, or IG-2D filter. The differences were highest when the number of variables used to build a model was 10 or 20.

We have also examined whether feature selection by a more sophisticated algorithm can lead to better results. For that we built lasso models using variables identified by t-test and selected *N* most important variables. Models built on variables selected by lasso consistently have a much higher OOB estimate of MCC than all models built using other methods, with highest MCC obtained for 20 variables. The picture changes when fully cross-validated estimate of MCC of models is considered. Models built using 10 or 20 variables selected by combination of t-test and lasso are still better than those obtained with other feature selection methods. However, when the number of variables is increased to 50 and 100 the quality of models built on variables selected by t-test+lasso procedure falls. In effect, the best models obtained with this method are no better than models obtained using simple t-test, and are significantly worse than models obtained by filters based on information gain.

It is interesting to note that models based on the features selected by lasso tend to overfit much more strongly than models built using simpler top N approach. The average difference between MCC computed using OOB approach and MCC computed in cross-validation is 0.21 for t-test+lasso, whereas for simple filters it is 0.16. Despite that difference, the correlation between MCC computed using OOB and MCC computed in a cross-validation is high - Pearson correlation coefficient between these results is 0.60 for all models generated for gene expression data sets limited to 145 patients.

#### Copy number variation.

The copy number data set contains significantly fewer informative variables than gene expression data sets. Moreover, models using this data have significantly lower predictive power, in particular when fully cross-validated approach is used, see Table 7. In particular, models built using variables identified by t-test are prone to overfitting in this case. The average MCC reported for OOB estimate for fully cross-validated models is 0.48, but it drops to 0.19 when measured by cross-validation. The lasso procedure does not help in this case, since, due to low sensitivity of t-test for CNV data set, there are only a few informative variables identified in each case, and lasso is not used at all. On the other hand, models built on variables identified with the help of filtering methods which use information theory fare much better. The average MCC for models built utilising IG-1D and IG-2D filtering is 0.26 and 0.31, respectively. The difference between IG-1D and IG-2D is small, but statistically significant (*p*-value < 0.000025). Interestingly, the models built on variables selected by IG-2D have lower OOB estimate of MCC than models built using all other feature selection models.

**Table 7:** Model quality measured with MCC coefficient for the CNV-145 data set

	OOB				Cross-validation			
FS metod	Top 10	Top 20	Top 50	Top 100	Top 10	Top 20	Top 50	Top 100
Stage 1								
T-test	0.566	-	-	-				
IG-1D	0.569	0.646	0.642	0.646				
IG-2D	0.460	0.484	0.491	0.492				
T-test + lasso	0.568	-	-	-				
Stage 2								
T-test	0.534	-	-	-	0.545	-	-	-
IG-1D	0.544	0.624	0.617	0.615	0.551	0.635	0.632	0.627
IG-2D	0.443	0.470	0.484	0.484	0.452	0.481	0.493	0.491
T-test + lasso	0.537	-	-	-	0.554	-	-	-
Stage 3								
T-test	0.476	-	-	-	0.189	-	-	-
IG-1D	0.575	0.582	0.583	0.582	0.248	0.257	0.258	0.260
IG-2D	0.502	0.511	0.514	0.514	0.279	0.301	0.308	0.306
T-test + lasso	0.510	-	-	-	0.193	-	-	-

#### Synergies between data sets

There are two possible sources of synergy in the current study: technical and biological. Firstly, gene expression was studied using different technologies, namely RNA sequencing and microarrays. What is more, RNA sequencing was represented by three different data sets measuring slightly different aspects of gene expression. Secondly, two different biological phenomena were measured, namely gene expression and copy number variation of genes. In the search of synergy we have analysed possible pairwise synergies between selected data sets. In particular, we have checked for possible technical synergy using MA-145 data set and all RNA-seq data sets. We have also measured possible technical synergy between data sets using different feature selection algorithms. In both cases no synergy was observed - models built using mixed sets of variables had lower cross-validated MCC than those achieved for at least one of the data sets under scrutiny.

More interesting results were obtained when biological synergy was examined. We explored possible synergies using variables selected from either G-145 or MA-145 data sets merged with variables selected from CNV-145 data set. For each feature selection method fifty highest scoring variables were selected from either gene expression data set. Then, the feature set was extended by all variables identified as relevant by the same method. Next, predictive models were built using the joint feature set.

The increase of MCC for mixed data sets with respect to the pure gene expression feature set were observed for both MA-145 and G-145 on the OOB level, see Table 8. In stage 2, where all variables were selected once, the increase was small but consistent and confirmed in cross-validation. Unfortunately, the results were not clear-cut in stage 3. Here, the increased MCC was again demonstrated in OOB estimate. However, the increase on the validation set was either non-existent or too small for clear confirmation. The highest increase, 0.005, which was still not significant, was obtained for the t-test + lasso method on the MA-145 data set, but this result may arise due to less overfitting in the model building stage and not due to genuine biological effects.

**Table 8:** Synergies between data sets

Data set	MA-145							
	OOB				Cross-validation			
Feature set	MA^50^	CNV	MA+CNV	Syn.	MA^50^	CNV	MA+CNV	Syn.
Stage 2								
T-test	0.693	0.537	0.693	-0.001	0.709	0.546	0.698	-0.011
IG-1D	0.729	0.617	0.755	**0.026**	0.737	0.632	0.765	**0.028**
IG-2D	0.721	0.484	0.740	**0.019**	0.731	0.493	0.750	**0.020**
T-test + lasso	0.808	0.536	0.804	-0.004	0.831	0.553	0.827	-0.004
Stage 3								
T-test	0.714	0.479	0.717	**0.004**	0.647	0.192	0.632	-0.015
IG-1D	0.747	0.583	0.764	**0.017**	0.661	0.258	0.662	**0.001**
IG-2D	0.730	0.514	0.740	**0.010**	0.655	0.308	0.656	0.000
T-test+lasso	0.808	0.506	0.825	**0.017**	0.648	0.194	0.652	**0.005**
	G-145							
	OOB				cross-validation			
Feature set	G^50^	CNV	G+CNV	Syn.	G^50^	CNV	G+CNV	Syn.
Stage 2								
T-test	0.714	0.537	0.720	**0.006**	0.716	0.546	0.725	**0.009**
IG-1D	0.766	0.617	0.780	**0.014**	0.778	0.632	0.786	**0.009**
IG-2D	0.743	0.484	0.747	**0.004**	0.756	0.493	0.757	**0.001**
T-test+lasso	0.848	0.536	0.853	**0.005**	0.865	0.553	0.868	**0.003**
Stage 3								
T-test	0.714	0.478	0.730	**0.016**	0.650	0.192	0.640	-0.011
IG-1D	0.747	0.582	0.786	**0.039**	0.661	0.258	0.662	**0.001**
IG-2D	0.730	0.511	0.767	**0.037**	0.650	0.308	0.650	0.000
T-test+lasso	0.808	0.506	0.858	**0.050**	0.655	0.194	0.655	0.000

Synergies between data sets displayed for two stages of the analysis for MA+CNV and G+CNV data sets. MA^50^ and G^50^ are sets MA-145 and G-145 data sets limited to top 50 variables, respectively. Cases, for which MCC for mixed model is higher then either of the components, suggesting possible synergy are highlighted in boldface

## Discussion

The small size of the data set, in particular the small number of objects in the less numerous class, presents the main challenge to the current study. The imbalance between survivors and non-survivors poses several difficulties and requires special care when designing the research protocol. In particular, it affects the design in two important aspects. The five-fold cross validation, with stratified selection of objects to training and validation samples, was used to ensure that training set contains sufficient number of objects for feature selection and for model building. We have observed a significant decrease of quality of models in three-fold cross-validation.

Secondly, due to the small number of samples the variance of the results was very high. Therefore, the high number of repeats in cross-validation was required to achieve good separation of results with different means. To this end, we have built 100 independent full cross-validation cycles for each data set and each combination of feature selection method and number of variables. This translates to construction of 500 independent Random Forest models, for each estimate of MCC. What is more, in stage three each model requires performing independent feature filtering. Filtering is very quick for t-test and IG-1D, but may take between roughly a minute for G-145 and MA-145 data sets, and a few hours for J-498 and T-498 data sets, when IG-2D is used. Consequently, the entire procedure is time consuming and requires substantial computational resources.

Finally, the ultimate cross-validated estimates of the model quality are most likely biased downwards, as demonstrated by negative correlation between OOB and validation set estimates of MCC. The influence of this effect may be estimated by converting the results of the entire cross-validation scheme to a new ensemble classifier, consisting of 500 independent models, each built using a different subset of objects and a different subset of variables. Each object has been set aside to the validation set once per full cross-validation loop, hence, we can have OOB estimate of performance for this ensemble of Random Forests. This measure may be a better estimate of the true performance of the classifier than that obtained as a simple average MCC over 100 repeats of the cross-validation scheme. The comparison of three estimates of MCC for MA-145 and G-145 obtained for models built using 100 variables is given in Table 9. One can see, that eight MCC estimates obtained for ensemble of forests for two different data sets and four different feature selection methods are fairly similar, despite larger differences both in OOB and cross-validated estimates. While we are not able to verify this conjecture within the framework of the current study, we may nonetheless treat it as a reasonable hypothesis.

**Table 9:** Three estimates of MCC

	Feature selection method							
Estimate type	T-test	IG-1D	IG-2D	T-test+lasso	T-test	IG-1D	IG-2D	T-test+lasso
	**MA-145 data set**				**G-145 data set**			
OOB	0.720	0.736	0.726	0.822	0.713	0.796	0.763	0.861
Cross-validation	0.665	0.674	0.670	0.643	0.653	0.672	0.661	0.638
Ensemble	0.702	0.727	0.711	0.699	0.689	0.705	0.712	0.698

Interestingly, analysis of the ensemble classifier shows that there are three classes of patients. The first, most numerous one, consists of the correctly classified patients for whom there is a very high (close to 100%) agreement between all member classifiers in the ensemble. Roughly 75% of objects in the smaller cohort belongs to this class. The second class consists of patients for which decision varies in different repeats of the cross-validation procedure. Roughly 15% of patients belongs to this class. Finally, roughly 10% of patients are incorrectly classified with very high agreement of decisions in different repeats of the cross-validation procedure. The existence of this group of patients shows the limits of predictive models for neuroblastoma based on molecular data.

## Conclusions

There are four main findings of the current study. Firstly, we have proposed a robust framework for evaluation of predictive models for small data sets, for which split of data between training and validation set may result in significant drop of accuracy due to insufficient sampling. This framework allows for the estimation of bias, which arises due to selection of variables that are best for model building in the context on the current sample. Application of this framework allows to project ranking of models estimated on the training set to the ranking on the validation set. The correlation between performance of models on the training set and validation set is 0.6, compared to correlation 0.04 obtained in the study by Zhang et al. [4] who presented the first analysis of the data sets examined in the current work. The cross-validated approach allows also to construct an ensemble classifier. In this higher-level ensemble of Random Forests, for each object a prediction made by elementary Random Forest within the cross-validation is treated as a single vote for the class of a given object. The estimate of MCC for this ensemble classifier is higher than the average MCC obtained in cross-validation. It is also our best guess for the performance on the new data for ensemble of classifiers developed with the presented methodology.

We have also examined the possibility of an increase of the predictive power of models built using combinations of data sets. The small synergy between copy number variation and gene expression was observed for the OOB estimate of MCC, but it was not confirmed in cross-validation. We hypothesize that this synergy could be confirmed if a larger sample size was to be used. This increase was observed despite very weak predictive power of models built on CNV alone.

Only a few genes were consistently discovered as most informative by all filtering methods for gene expression data sets, however, those for which all methods were in agreement were previously identified as related to neuroblastoma. Interestingly, the average gene expression level for the genes commonly identified as relevant in microarray experiments and RNA-seq was identical to those identified as the most relevant by microarrays only. On the other hand, the genes that were identified by RNA-seq only had a significantly lower average expression level. This result aligns with previous findings that RNA-seq allows to identify significant genes with lower expression levels due to higher resolution and lower noise level of the method in comparison with microarray experiments [49].

Finally, despite a divergence of genes identified by different methods for feature selection, models built using expression of these genes gave similar results, with slight but regular advantage of filters based on information gain. The more aggressive feature selection, with the help of lasso method, gives best results when low number of variables are used, but overfits for larger data sets. Both filters based on the information gain show their advantage for the CNV data set, where they are more sensitive and allow for building better models. What is more, the results obtained for the CNV data set demonstrate the utility of feature selection that takes into account interactions between variables. The IG-2D filter was most sensitive for this data set, and, what is more, the models using variables found by this filter were best for this data set.

## Reviewers’ comments

### Reviewer’s report 1: Lan Hu

**Summary** There are technical merits in the study. However the manuscript language and organization need to be much improved for clarity. There are obvious grammatical errors that should have been corrected by the authors. The technical description was unfortunately sloppy and difficult to follow.

**Reviewer recommendations to authors**

1. Correct the language issues and clean up the manuscript. Here are a few examples of grammatical improvements: ‘To this end’ → repetitive occurrences of this phrase with no clear benefit

‘In the current study two cases are explored, namely *S*= and |*S*|=1 ’ → missing ‘1’ between ‘=’ and ‘and’? ‘are to numerous to be useful for further analysis’ → ‘are too numerous to be useful’...

Authors’ response: *We have reached for external help with grammar and edited the text to improve readability. In particular, we have corrected all of the issues raised above.*

2. Need to improve the technical description. Authors should pay more attention to technical terms.: For example, on page 14, line 62 says ‘the DNA expression was studied using different technologies...’. ‘DNA expression’ is not a correct term, but ‘gene expression’.

Authors’ response: *We have checked the manuscript and corrected all cases that we were able to identify. In particular, we have corrected the term mentioned above*

3. Page 10, the last paragraph of “Gene expression” section. What is the unit of ‘average intensity’ of gene expression reported in microarrays and RNA-Seq? The authors made a simple comparison between two sets of numbers from the two platforms to conclude that ‘higher signal to noise ratio in RNA-seq experiments can reveal strong biological signal in weakly expressed genes’? How?

Authors’ response: *Both microarrays and RNA-seq are used to measure gene expression, but due to the differences in technology and experimental protocol, the numerical value of gene expression is valid within a single experiment/technology, but not directly comparable between technologies. In particular, in the case of microarrays the signal is the logarithm of the recorded fluorescence intensity, which in turn corresponds to the number of transcripts hybridised to the sondes. In the case of RNA-seq, the signal is the logarithm of the normalised number of reads mapping to a particular gene. We have added an in-depth explanation of this issue to the description of data in the Methods section*

*Since the same signal is measured in different units, one cannot compare them directly. However, one can use the genes considered as most relevant by both experimental techniques as a reference. To this aim, one can compute the average strength of signal for this reference set in both technologies. Then it is possible to examine whether significant differences can be found between the average expression of genes in the reference set and the expression of genes in the sets unique to the particular technique. It turned out that the signal is identical in both cases for microarrays, but it is significantly lower in the set of genes unique for the RNA-seq than in the reference. Hence, we propose the hypothesis that this may be connected with the claimed higher sensitivity/resolution of RNA-seq. We have reformulated the fragment describing this observation.*

### Reviewer’s report 2: Tim Beissbarth

**Reviewer summary** In the manuscript the predictive power of a neuroblastoma data set is analyzed based on omics measurements on three different levels, i.e. genetic variants, copy number variations and gene expression. An extensive cross-validation and feature selection pipeline is set up. The authors can show that entropy performs a bit better in the feature selection process than t-test and that combining information of the three different data sets gives an improved performance. Of course the method of combining the different data sets is a bit naive - with genetic information there are often millions of variants and the levels of the data are ideally discrete (0,1,2), the number of copy-number variations is typically much lower and there should also be a discrete distribution, gene expression levels usually have a more-or-less continuous log normal distribution (or negative binomial distribution for RNA-Seq data) and there are usual several thousands of genes expressed. Of course these different distributions and numbers of variables in the individual data sets leads to biases in the feature selection when these data are combined. Nevertheless, I think this is a valuable work and can lead to a better discussions and to improvements of the methods in the future.

Authors’ response: *We agree with reviewer that feature selection for a model that uses data sets obtained with completely different methodologies and describing different biological processes may be difficult and involve several biases. However, the current study does not involve SNP data, with its very high dimensionality and very strong correlation structure and discrete values. Hence, the problem is limited to combination of data on copy number variation with data on gene expression.*

*This task is significantly easier. While CNVs are discrete in the most basic level* (i.e. *a genome of a single cell certainly contains a discrete number of copies of a particular gene), in practice this data is a continuous variable, due to following factors: the biological material contains a mixture of cells at different stages of tumor development, the experimental measurement procedure is inherently noisy, the experimental measurement corresponds to the colour that arises from different intensity of two dyes in a particular sample; the proportion of dyes is encoded as a real-valued number, the logarithm of this number is used in the final data set. The convolution of these factors results in a data set comprising of real-valued numbers between -3 and 3, in most cases conforming to the normal distribution.*

*We have tried two methods for combination of data sets. In the first one, we simply merged two full data sets before applying feature selection. Unfortunately, the signal from the CNV data set was much weaker than from either MA-145 or G-145 data sets. In effect, after application of Bonferroni correction, very few variables describing CNV were found in the top 100 variables.*

*Therefore, we decided to use the method described in the article, without describing the failed attempt with the simpler method mentioned above. The advantage of the method finally used in the article is that all variables that were discovered as relevant for CNV data were then used in joint models. The disadvantage is that possible synergies between variables not identified as relevant in separate analyses could improve models. However, such synergies should be detectable by our two-dimensional analysis performed on the joint data set. Unfortunately, none were observed. We have extended subsection* “Aggregation of data sets” section “Methods” section *to discuss this issues.*

**Reviewer recommendations to authors** - please discuss the different statistical properties and distributions of the different measurement techniques.

Authors’ response: *We have added a discussion of the statistical properties of the data sets obtained with different measurement techniques to the description of data, in the new the sub-subsection* “Statistical properties of gene expression and CNV data”, “Data”, “Methods” sections

- please describe the different preprocessing pipelines for the different data types better and discuss the steps to make these data statistically comparable

Authors’ response: *We have added description of data preprocessing in the new sub-subsection* “Data preprocessing”, “Data” and “Methods” sections

- discuss availability of source code.

Authors’ response: *The code for computing information gain and estimate od the statistical significance is available as the open source module MDFS deposited on CRAN. The scripts for performing analyses are available on request. We have added an appropriate note in the section “Availability of supporting data.”*

### Reviewer’s report 3: Dimitar Vassilev

**Reviewer summary** The submitted text for paper is of definite interest focussing the domain of problems concerning the prediction of survival time in neuroblastoma cancer studies. Among the given approaches for features selection there is some advantage of the models based on information entropy as compared to the pure statistical (t-test) and machine learning predictive models. Despite of the fact that obtained results are not with drastic improvement from some previous studies of the same type (Zhang et al. 2015) there are some valuable outcomes in the submitted work. First obvious merit is the capacity of authors in using various models with various features selection, with various data sets, organized in a of framework. The second technical achievement of the work is suggesting ways of increasing of the predictive power of the models. And the third benefit of the work is the comparison of prognositc models for integrated sources of information from gene expression (GE) and copy number variants (CNV) which has a potential to give some quality in discovering more genes, strongly related to survival time. Although, there are some obvious obstacles to obtain results of good value - strongly connected with the data by itself and less connected with the models and approaches used. The provided data at first sight perhaps is good for a research publication but it is obviously very limited in number and unbalanced. The set of 145 patients: split in 107 and 38 by surviving trait is obviously not enough for applying such set of methodological tools - in particular in classifying the data and making predictions by machine learning. This criticism could be related also to CAMDA challenge which provided the data and the tasks for analysis, but nevertheless the data is as it is and the results from the study are related to this particular data and not to other one. Aside with that there is obvious data dependance, which in particular influenced the analysis when the data set is split in smaller sets aiming at better tuning of features selection. The other problem which is directly subjective to the authors is the strict use of models without any larger comparative explanation - why that has been done. Yes, the models are explained by themselves but why particularly are used needs more. Such is the case with Mathew’s Correlation Coefficient (MCC) which by literature is almost ideal binary classifier - but definitely it depends on the data and is not too much universal. Also the Random forest approach as predictive model is well explained by why the machine learning methodology in the submitted material is based particularly on the Random Forest. I would suggest authors to throw more light on the reasons they have selected those approaches and possibly this will explain some of the not very eloquent results as small synergy between CNV and GE. I think that the good think in the submitted work is the not bad implementation of the information gain method for identification of informative variables. Such a method is not pure statistical and to my concern methods from informatics will have some advantage in such studies in particular where is a desperate need for avoiding the data dependance as in the submitted material. My opinion is that the definite technical outcome of the paper is that there is some room for improving the models for survival time prediction by using different models, based on different feature selection schemes. Apart of these my remarks and criticisms I would to recommend the submitted material to be published after a careful revision.

**Reviewer recommendations to authors**

The submitted work is of a good quality and I would encourage it publishing. There are several obvious merits of the work mostly connected to the technical aspect of the analysis. The use of different models for integrative analysis of the survival time for gene expression and copy number variants in neuroblastoma cancer studies. The models are based on different approaches for feature selection by using statistical, informatics and machine learning methods. The study provides also a framework for cross-validation protocol, that includes feature selection within cross-validation loop and classification using machine learning. The dependence of results on feature selection is assessed by different models. All these set of models, approaches, protocols, etc give obvious merits to the study. Aside with that there are definite problems obtained and exposed in the study.

Authors’ response: *We appreciate the appreciation of the merits of our work by reviewer, and we agree that there were some aspects of the study and its description that could be improved.*

**Reviewer:** The first major problem is the given data set. It is definitely too small and unbalanced. There are also some hidden dependencies in the data, in particular when it is split in smaller subsets for better feature selection tuning. All these facts affect the subsequent analytical approaches. The major problem there is possibly the unbalancedness of the data - 107 vs 38 cases for survival time prediction. All these facts affect the subsequent analytical approaches.

Authors’ response: *We agree that the data set is small and unbalanced and it poses difficulties for model building and validation. In particular, the small size of the data set and principally the minuscule number of cases in one class result in a very large variance of results. This had a decisive influence on the setup of the study. We have used 5-fold cross validation since the models built within 3-fold cross validation gave significantly worse results also on the OOB level. The large number of replications of cross-validation runs (one hundred) was necessary for reducing the standard deviation of the means to reasonable levels - the standard deviation of MCC for MA-145 data set was about 5 times higher than for MA-498. Unfortunately, this was an external constraint of the study, the organisers of CAMDA provided such datasets and no more data was available.*

**Reviewer:** First is the classification methodology - the popular for unbalanced data sets Mathews Correlation Coefficient obviously is not the best solution for this particular data set.

Authors’ response: *We don’t agree with the reviewer’s opinion on MCC, and we believe that this is a very good metric. A thorough explanation of the properties of MCC was given by Powers in a highly cited article from 2011 (Powers, D.M.W., J. Mach. Learn. Technol., 2(1), 37–63). What is more, the MCC was used by Zhang* et al. *in the original study with RNA-seq and microarray analysis of neuroblastoma. Hence MCC was the natural choice for comparison with the original study. Nevertheless, we are grateful to reviewer for raising this issue, since it clearly has shown a need for a better justification for application of MCC for comparisons between models. We have added the explanation that supports our selection of MCC in the subsection* “Comparisons between models” section “Methods” sections

**Reviewer:** The same is the case with Random forest predictive value classifier as a machine learning approach. The results obtained by using those approaches can be related as methodologically poor and the authors need to elucidate why. Why these approaches are used, can they be compared to another ones of the same class, are there any other opportunities. Obviously the aim of the authors to improve the results given by Zhang et al. (2015) somehow limits and does not present author’s capacity in choosing the best combination of models and defining the reasons of the potential results. There is an obvious need for additionally explanation of the efficiency of the selected models in the study. I think that for such data will be difficult to obtain drastically improved results, but it will be worth to present in the submitted material the reasons of using such models.

Authors’ response: *We don’t agree with the reviewer’s opinion that selection of the Random Forest is a bad one, and we have several reasons for that. First, Random Forest is generally a robust classification algorithm, that has been used for diverse classes of problems usually with very good results. Indeed, there is a recent paper (Fernandez-Delgado* et al. *J. Mach. Learn. Res 15(1), 3133–3181) devoted to testing multiple algorithms on numerous publicly available datasets. To be more specific, 179 algorithms belonging to 17 broad families of algorithms were tested on 121 datasets. The best overall results were achieved by algorithms belonging to the Random Forest family. What is even more important, RF algorithms not only achieved highest average rankings, but also rarely failed - for most problems they achieved results that are close to the best result obtained for the particular problem by any algorithm. Secondly, the RF is actually quite well suited for gene expression studies. There are well cited papers claiming that better classification accuracy for microarray data can be obtained with SVM, however, even when the results obtained with SVM were better, the differences were small in most cases. What is more, there are some newer papers showing outcomes with opposite results, cited in the modified text. The third reason is the computational cost. SVM requires extensive computations to obtain best parameters for particular problems. This includes selection of the appropriate kernel function and derivation of best parameters for the kernel. For proper validation within the framework of the current study, all these computations should be performed within cross-validation loop, which would be prohibitively expensive computationally. This problem does not exist for Random Forest - sound results are usually obtained with default selection of parameters. Hence, no optimisation is required, even though in some cases, one can achieve improved results by tuning the number of variables considered in creation of split. Finally, the aim of the study is not achieving the best possible classification result, rather the examination of the hypothesis that a significant improvement of models can be achieved by synergy between data describing different biological phenomena. Random Forest is a good choice of an algorithm for discovering non-additive and non-linear effects. Due to its robustness, one can be assured that it will be able to use the information - if the information is available at all. We have extended the “*Methods*” section to better explain the rationale for the selection of Random Forest as the classification algorithm in our research protocol. In particular, we have rewritten and extended the subsection “*Predictive models*” section “*Methods*” section*

**Reviewer:** This will throw more light on the problems with the small synergy between different sampled datasets both in technical and biological context. The use of data from combined gene expression (GE) and copy number variants (CNV) at first sight bears more potential for the predicting power of the models, but unfortunately the limited size of the dataset have a stronger influence. This affect obviously the discovery of bigger number of important for survival time genes. Here need to be emphasised the applicability in such studies pure statistical, machine learning and informatics approaches based on features selection. The use of bit more successful model for informative variables detection as the Informative gain approach possibly can provide a background for better choice of the models for data integration and feature selection at all.

Authors’ response: *We were thrilled by possibility of synergies between CNV and gene expression, and this is why we undertook the study. However, in hindsight, we think that it is actually unlikely to observe such synergies in the large scale, for a simple reason. Most of the CNV’s contribution to the functioning of the cellular machinery should be already reflected in the gene expression profiles. Deletion of certain genes or multiplication of others should be reflected in lower or higher expression levels respectively. Therefore, it should be visible in the expression patterns. So, even if CNV’s contribute to the development of cancer, they do it by modifying gene expression levels. One should also remember that the development of cancer is a random evolutionary process, and the final outcome depends on the balance between multiple factors. In particular, the pace of development of mutations and the pace of development of immune response to cancer. Therefore, one can expect that prognosis of survival based on CNV should be less precise than one based on gene expression - simply because there are more factors modifying the response to CNV than to gene expression. Having said that, we don’t feel competent enough pursue this line of reasoning.*

*On the other hand we believe, that rigorous methods for identification of informative features involved in synergistic interactions can be useful for integration of variables from different sources.*

**Reviewer:** It will be worth to see the authors comment on comparison of models based on statistics, on machine learning and informatics. I think that a sort of combining such approaches may have good influence on the results for such studies. Aside with all those remarks and criticisms, I would dare to recommend the submitted material to be published after a careful revision.

Authors’ response: *We think that meaningful comments could be made if better results were achieved. In such a case, wider comparison of different approaches could be made. We agree with the reviewer that, in principle, this could be an interesting analysis to do – in particular if some synergies were found. Unfortunately, it seems that very little synergy can be gained from combining CNV and gene expression and this picture is unlikely to change when other methods are used. Hence, we do not believe that such an analysis would be worthwhile for the current study. Therefore, we would rather prefer not to venture into further reaching comparisons. This would require significant effort to perform similar analysis with different set of tools and then comparison of results achieved. Otherwise such comparisons would be purely speculative.*

**Reviewer: Minor issues**

1. The language of the paper is a bit heavy and obscure.

Authors’ response: *We have strived to improve the language in the current submission.*

2. There is dedicated to much space in a meticulous explanation of the used approaches but not an explanation for their use in this case study in particular. I would recommend to make a sort of comparative explanatory analysis of the used models with particular reasons to the study.

Authors’ response: *We have extended the “*Methods*” section to include some explanation why such choices were made. Most answers to previous comments cover that. We have also modified the first paragraphs of the “*Methods*” section to stress the reasons behind the choices made.*

3. The abstract is written in a bit shy manner. There are lot of sentences with “...slightly significant...”, “...slightly different...” The results should be presented as they shortly discussing the reasons for such outcomes.

Authors’ response: *We believe that abstract is not really that shy since only marginal results were obtained for the main goal of the study, namely discovering the synergy between data from different experimental techniques for better predictions of survival in neuroblastoma. On the other hand, we were not shy when describing the main strength of the study, namely the development of the robust predictive methodology. We would prefer to stay with the modest approach, risking being too shy rather than too optimistic.*

4. I would recommend also to reduce the length and complexity of the sentences in the text. Authors’ response: *We have strived to improve the language in the current submission, in particular we used shorter and simpler sentences where possible.*

### Second round of reviews: Reviewer’s report 1: Lan Hu

**Reviewer comments to Authors** The authors have taken great effort answering the reviewers’ comments and recommendations. As a result, the paper is much improved from the previous version.

**Minor issues:**

1. It would be helpful to include the stats of survival status of patients in each of 498 and 145 datasets.

Authors’ response: *We have added required information at the end of subsection Data.*

2. page 5, line 50:

two different Affymetrix matrices -> two different Affymetrix platforms.

Authors’ response: *We corrected nomenclature in the requested manner.*

### Second round of reviews: Reviewer’s report 2: Dimitar Vassilev

**Reviewer comments to Authors** I am satisfied with the answers. Definitely there remain some open questions in the choice and validation of the machine learning methods used in the study - but this needs larger comparative approach and very possibly larger dataset.

Authors’ response:*We agree with the reviewer that a large comparative study for comparing efficiency of different modelling approaches would be worthwhile.*

**Minor issues:**

I accept the corrections made by the authors.

## Abbreviations

aCGH: Array comparative genomic hybridization
CAMDA: Critical assessment of massive data analysis
CNV: Copy number variation
FS: Feature selection
G: General referense to gene data set
G-145: Gene data set limited to 145 patients
G-498: Gene data set for 498 patients
GE: Gene expression
IG: Information gain
IG-1D: One dimensional relevance test based on information gain
IG-2D: Two dimensional relevance test based on information gain
J: General reference to junction data set
J-145: Junction data set limited to 145 patients
J-498: Junction data set for 498 patients
lasso: Least absolute shrinkage and selection operator
MA: General reference to microarray data set
MA-145: Microarray data set limited to 145 patients
MA-498: Microarray data set for 498 patients
MCC: Matthews correlation coefficient
OOB: Out of bag
RNA-seq: RNA sequencing
RF: Random forest
SVM: Support vector machine
T: General reference to transcript data set
T-145: Transcript data set limited to 145 patients
T-498: Transcript data set for 498 patients
